# Comparing the effectiveness of extracorporeal shockwave therapy and myofascial release therapy in chronic pelvic pain syndrome: study protocol for a randomized controlled trial

**DOI:** 10.1186/s13063-023-07633-1

**Published:** 2023-10-18

**Authors:** Ningqing Huang, Zhi Qin, Wudong Sun, Kaiming Bao, Jingxian Zha, Peng Zhang, Panpan Feng, Xiaojun Zhao, Mengqian Liu, Jinjun Shi, Ming Ma

**Affiliations:** 1 Department of Physical Medicine and Rehabilitation, Southeast University, Nanjing, Jiangsu Province People’s Republic of China; 2 Department of Radiology, Southeast University Zhongda Hospital, Nanjing, Jiangsu Province People’s Republic of China; 3 Department of Rehabilitation, Southeast University Zhongda Hospital, No. 87, Dingjiaqiao, Gulou District, Nanjing, Jiangsu Province People’s Republic of China; 4 Division of Biokinesiology and Physical Therapy, University of Southern California, Nanjing, Jiangsu Province People’s Republic of China; 5 Department of Obstetrics, Southeast University Zhongda Hospital, Nanjing, Jiangsu Province People’s Republic of China; 6 Nanjing Institute of Physical Education, Nanjing, Jiangsu Province People’s Republic of China; 7 Southeast University Zhongda Hospital, Nanjing, Jiangsu Province People’s Republic of China

**Keywords:** Chronic pelvic pain syndrome, Extracorporeal shockwave therapy, Myofascial release therapy, Electromyography, Autonomic nervous system

## Abstract

**Background:**

Chronic prostatitis/chronic pelvic pain syndrome is a highly prevalent syndrome. Previous studies showed that extracorporeal shockwave therapy and myofascial release therapy could improve the quality of life in patients with chronic prostatitis/chronic pelvic pain syndrome (CP/CPPS). Theoretically, combined therapy with extracorporeal shockwave therapy and myofascial release therapy will likely have significant advantages in treating CP/CPPS. We, therefore, present a protocol for conducting a well-designed randomized controlled trial to compare the efficacy and safety of each therapy.

**Methods:**

The proposed study will be a three-group randomized control trial (RCT) design that includes 150 participants from Zhongda Hospital Affiliated to Southeast University, with equal allocation of participants to the three intervention groups. The study duration will be 8 weeks, which includes a 4-week treatment period and a 4-week follow-up period. The primary outcome will be the changes in surface electromyography (sEMG) assessment and National Institutes of Health-Chronic Prostatitis Symptom Index (NIH-CPSI). The secondary outcomes will include the changes in three-dimensional quantification, shear wave elastography (SWE), and sympathetic skin response (SSR) testing. Assessments will be conducted before the intervention (T0), before the 5th intervention (T1), immediately after the 8th intervention (T2), and the 4th week after the end of the 8th intervention (T3).

**Discussion:**

This trial will compare the differences in efficacy between single extracorporeal shockwave therapy, single myofascial release therapy, and combined therapy to select the most appropriate treatment option for patients with CP/CPPS. The possible pathogenesis of CP/CPPS would also be analyzed by comparing the intercorrelation between each objective and subjective measurement (NIH-CPSI score, sEMG, SWE, SSR).

**Trial registration:**

The name of the registry: Extracorporeal Shockwave and Myofascial Release Therapy in Chronic Pelvic Pain Syndrome.

Registration number: NCT05659199. Date of registration: December 2022.

**Supplementary Information:**

The online version contains supplementary material available at 10.1186/s13063-023-07633-1.

## Background

Chronic prostatitis/chronic pelvic pain syndrome (CP/CPPS) is a kind of psycho-neuromuscular disorder [1, 2]; the global prevalence of CP/CPPS is between 9 and 16% [3]. In China, the prevalence of chronic prostatitis/chronic pelvic pain syndrome is about 3.9% [4]. Predisposing factors for CP/CPPS include sedentary behavior, excessive masturbation, frequent sexual intercourse, high psychological stress, anxiety, and depression [5]. With the increase in sedentary behavior during the COVID-19 pandemic, the prevalence is likely to be underestimated [6]. Chronic non-cyclical pelvic pain and discomfort in the pelvic region caused by CP/CPPS usually lasts for at least 3 of the past 6 months [7] and is often accompanied by lower urinary tract symptoms [8], psychosocial impairments, sexual dysfunction, postejaculatory pain [9], and pelvic floor muscle (PFM) pathology change [10]. The specific symptoms are pain in the groin and penile root, pain during erection, frequent urination, and a burning sensation in the urethra [11, 12]. It is now well-accepted that pelvic region pain can impair patients’ psychological state and quality of life [13]. Epidemiological studies have shown that psychosocial changes such as anxious depressive states and stress reactions [14] caused by CP/CPPS can further exacerbate pain symptoms severely [15]. Also, somatization symptoms, depression, anxiety, and pain catastrophizing were associated with physical HRQoL 12 months after treatment [16]. For this reason, it is vital to develop effective interventions to alleviate pain caused by CP/CPPS.

One of the causes of pelvic pain may be the spasm of PFM and impaired ability to relax [17]. When palpating the pelvic floor muscles of the patients, there are apparent tender points. The tissue at the tender point differs from the surrounding muscle tissue, with a certain degree of sliding limitation and ischemic sclerosis. Myofascial release therapy can potentially reduce muscle stress, create flexible fascial structures, and improve vascularization and innervation of the affected region [18]. The fascia trigger point is less painful, and tissues are softer after myofascial release therapy. However, patients may experience physical and psychological discomfort because the treatment will pass through the rectum. Palpation by the rehabilitation therapist is one of the most common procedures used to determine the muscle state of pelvic floor. However, this method is too subjective. So, we will evaluate pelvic floor muscle surface electromyography to determine if CP/CPPS patients commonly have elevated pelvic floor muscle tone at rest.

The existing body of clinical research on CP/CPPS suggests that extracorporeal shockwave effectively reduces pain and urinary tract discomfort and improves patients’ quality of life, with good long-term outcomes [5, 19–21]. To explain this result, Hung-Jen Wang [22] proposed the sequence of central pain processing, and the nociceptive effect at DRG was retrogradely attenuated, followed by the decrease of COX-2 and NGF expression in the prostate after Li-ESWT, which then provides long-term pain relief. However, they have rarely explained the role of extracorporeal shockwaves in changing pelvic floor muscle tone and elasticity. On the other hand, we found the fact that some reports on cardiovascular research show extracorporeal shockwave can effectively relieve muscle spasm in patients with hemiplegia [23–25], which may help relieve the increased pelvic floor muscle tone in patients with chronic pelvic pain syndrome [25–27].

Previous studies reported that abnormal autonomic nervous system (ANS) might correlate with chronic pelvic pain [28]. However, to date, few objective assessment indicators have been used for evaluating autonomic nervous function in patients with CP/CPPS. This study employs, for the first time, evaluation of sympathetic skin response. The main reason for choosing this measurement is that SSR can reflect the functional state of the postganglionic fibers of the sympathetic design, although its effectiveness in evaluating CP/CPPS patients has not been systematically investigated.

The primary aims of this research are threefold: (1) to identify more relatively effective interventions for improving pain symptoms in CP/CPPS patients; (2) to ascertain the correlation between PFM elastic modulus and tenderness symptoms; this study may find a more objective method of assessing efficacy; and (3) to determine the correlation between the intensity of the sympathetic skin response and the patient’s symptoms and to explore other possible pathogenetic mechanisms.

### Composition of the coordinating center and trial steering committee

The coordinating center comprises clinicians and therapists from the Department of Rehabilitation and the Department of Ultrasonography of Zhongda Hospital. The principal therapists will supervise this trial and be responsible for the medical responsibility of patients. There is no trial steering committee (TSC) for this trial.

### Study setting

This trial (NCT05659199—ClinicalTrials.gov) is a monocenter randomized controlled trial with three groups conducted in Department of Rehabilitation and the Department of Ultrasonography of Zhongda Hospital, Nanjing, Jiangsu, China. Zhongda Hospital is a teaching hospital, where the total number of outpatients annually is 2.03 million. The Zhongda Hospital has a dedicated Pelvic Floor Health Management Centre to assess, treat, and follow-up patients with pelvic floor dysfunction. Patients admitted to the Pelvic Floor Health Management Center include those seeking postpartum pelvic floor rehabilitation, those with chronic pelvic pain syndrome, and others with pelvic floor dysfunction. Treatment provided by the Pelvic Floor Health Management Center includes pelvic floor biofeedback electrostimulation, pelvic floor magnetic stimulation, extracorporeal shockwave therapy, and myofascial release therapy. Myofascial release therapy is a kind of manipulation therapy. Between 2020 and 2023, more than 1000 patients with pelvic floor dysfunction were treated at the Pelvic Floor Health Management Center. In this trial, the subjects included will be randomly assigned to the extracorporeal shockwave, myofascial release, and combined therapy groups.

### Composition of the data monitoring committee

IEC (Independent Ethics Committee) for Clinical Research of Zhongda Hospital, which is independent of the sponsor and competing interests, will be responsible for monitoring the data of the trial.

### Objectives

Thus far, there is a lack of well-designed randomized controlled trials to compare the efficacy of the two treatments, as well as a unified objective evaluation index. Furthermore, these assessment procedures (mainly based on palpatory findings) typically suffer from issues of reliability and validity, although there has been a recent increase in the literature on this topic. The intervention protocols were seldom standardized and more frequently semi-standardized. The overall quality of evidence was judged as “very low.” All the RCTs did not follow the blinding procedures, certainly for participants and personnel.

## Methods and analysis

### Study design

**Fig. 1:**
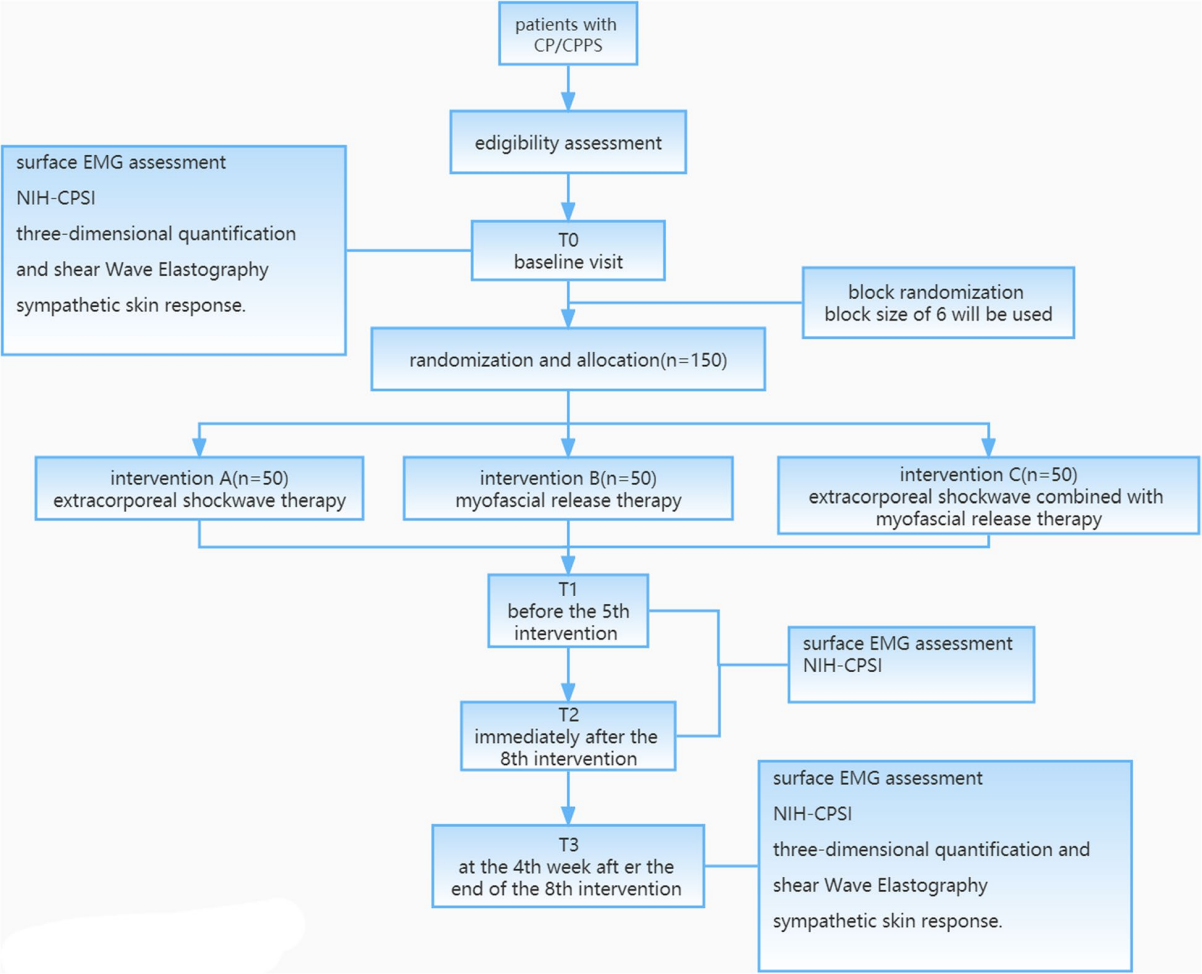
Research flowchart

The proposed study will be a three-group randomized control trial (RCT) design that includes 150 participants from Zhongda Hospital Affiliated to Southeast University, with equal allocation of participants to the three intervention groups. The research flow chart is shown in Fig. 1. The reporting of this protocol is strictly based on the Standard Protocol Items: Recommendations for Interventional Trials (SPIRIT), which is found in Additional file 1.

General information, medical/treatment history, and pre-assessment results of each participant will be confidentially collected by the research assistant before the initial intervention.

Assessments will be conducted before the intervention (T0), before the 5th intervention (T1), immediately after the 8th intervention (T2), and at the 4th week after the end of the 8th intervention (T3), which include the surface EMG evaluation of pelvic floor muscles, NIH Chronic Prostatitis Symptom Index (NIH-CPSI), three-dimensional quantification, shear wave elastography, and the sympathetic skin response.

The primary outcome measurements will be performed at T0, T1, T2, and T3. Secondary outcome measurements will be carried out at T0 and T3.

The SPIRIT figure is displayed in Table 1.

**Table 1:**
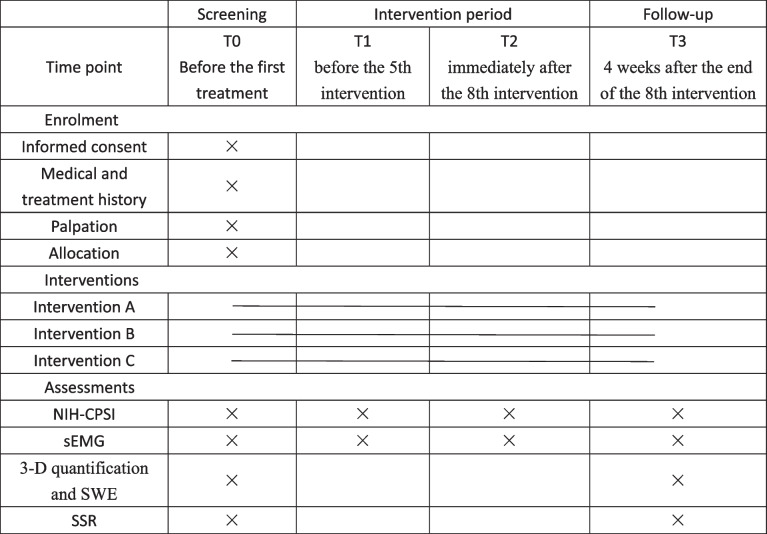
SPIRIT figure

### Randomization

One hundred fifty patients with CP/CPPS were selected and will receive treatment in Zhongda Hospital affiliated to Southeast University from September 2022 to September 2023.

### Sequence generation

Randomization 1:1:1 is done using https://www.sealedenvelope.com/simple-randomiser/v1/lists with varying block randomization with a block size of 6. No stratification is used.

### Concealment mechanism

The randomization sequence will be placed in sequentially numbered, sealed, and opaque envelopes. Sealed envelopes with extracorporeal shockwave therapy (1), myofascial release therapy (2), and combined therapy (3) will be made beforehand for the included trial participants by an outsider, and investigators will not know the content of the envelopes. After the patient signs the consent form, an envelope will be opened to assign an intervention for the patient.

### Implementation

The randomization will be done according to the sealedenvelope.com algorithm. The participants will be enrolled after an outpatient visit, and on the following day, the participants will show up for the informed consent. The primary investigator will open the sealed envelope at the end of the baseline examinations.

### Eligibility criteria

Patients with hormone abnormalities are excluded because thyroid dysfunction and testosterone may affect prostate function and myofascial status.

Participants will be screened using the following criteria.

Inclusion criteria:

1. According to the National Institutes of Health classification of prostatitis [29], all male patients meet the diagnostic criteria for chronic prostatitis type IIIB/chronic pelvic pain syndrome (pain in the bladder, groin, perineal area, genitalia or lower abdomen with no significant abnormalities on urological examination);
2. Patients are aged 20–40 years;
3. Patients with chronic pelvic pain lasting over 6 months;
4. Patients sign the informed consent form.

Exclusion criteria:

1. Patients with significant coagulation disorders, perineal anatomical abnormalities, hormonal abnormalities, and neurological abnormalities;
2. Patients with an apparent cause of pelvic pain, such as a history of previous surgery, chronic infection, trauma, prostatitis, and epididymitis;
3. Patients receiving other treatments during the study;
4. Patients with any urethral pathology;
5. Patients who have had closed lumbar injections and previous lumbar surgery within 6 months;
6. Patients with other conditions causing pelvic pain;
7. Patients with contraindications to physiotherapy;
8. BMI > 22.9

Termination and shedding criteria:

1. Other serious illnesses occur in the course of treatment;
2. Poor patient adherence and failure to complete the full course of treatment.

### Criteria for discontinuing or modifying allocated interventions

Patients receiving other treatments during the study will be excluded as exclusion criteria, but rehabilitation interventions will still be provided. Participants who experienced severe adverse events during the intervention will discontinue treatment, and the data that have been collected will be used for subsequent data analyses.

### Strategies to improve adherence to interventions

Three groups of participants will be treated by specially trained therapists and the intervention process will strictly follow the study protocol. If the participant is not treated and evaluated at the specified time interval, the primary investigator will contact the participant to ensure that the intervention is proceeding rigorously. If the participant continues to be absent from the treatment as needed or firmly withdraws from the study, the relevant data that have been collected will be used for subsequent data analyses.

### Recruitment

When all criteria for participation are met, the patient can consider participation and ask questions to any therapist. If a patient decides to participate in the trial, the patient signs an informed consent form. Subsequently, the patient will be allocated to one of the three groups. Participants cannot switch from the extracorporeal shockwave, myofascial release, and combined therapy groups. However, a patient is allowed to withdraw from the trial without providing a reason for withdrawal and still receives usual care.

### Intervention

Prior to treatment, all patients undergo palpation by the rehabilitation therapist, which includes palpation of the internal muscles of the perineum and rectum using a massage wand with the position of bladder lithotomy (including but not limited to the bulbocavernosus muscle, deep transverse perineal muscle, anal raphe and recto-urethral muscle). With the patient lying supine and laterally, palpate the abdomen, back, buttocks, and inner thigh muscles. Researchers will record the location of the patient’s fascial trigger points, the nature of the pain, and the number of tenderness points recorded.

#### Intervention A—extracorporeal shockwave group

Patients will be treated with extracorporeal shockwave therapy (ESWT) with bladder lithotomy position, twice per week for 4 weeks, 3000 individually with a maximum total energy flow density of 0.25 mJ/mm^2^, at a rate of 3 Hz on each occasion. Extracorporeal shockwave (RUIDI.SWT001, Shenzhen, China) can provide some physical spark wave energy that will be delivered by the probe. The water sac probe will be moved slowly over the groin, perineum, and crura of the penis.

#### Intervention B—myofascial release group

Based on the palpation findings, the pressure was applied at 1 kg/cm^2^ (within the patient’s tolerable range depending on the individual) to the points where patients had a VAS pain score of 4 or more during palpation. At tenderness, intermittent pressure will be applied for 180 to 210 s until the muscle relaxes.

#### Intervention C—combined therapy group

In addition to routine palpation, the combined intervention group will be treated with extracorporeal shockwave and myofascial release therapy in a format identical to that of interventions A and B.

All the treatment will be administered once a day, 2 times a week, at no less than 2-day intervals for 4 weeks, for a total of 8 sessions.

#### Primary outcome measures

Effectiveness assessment indicators:

1. Pelvic floor muscle surface electromyography (sEMG).

Pelvic floor muscle sEMG values will be collected using the Glazer pelvic floor muscle sEMG assessment (VISHEE SA9800).

#### Pre-resting sEMG

Patients will be required to rest for 1 min to relax the abdominal, gluteal, and perianal muscles fully. Then, we will measure the amplitude and coefficient of variation of pelvic floor muscle resting tone (coefficient of variation = standard deviation/mean). Mean sEMG values greater than 4 μV suggest that the pelvic floor muscle tone is elevated and the muscle is in a state of spasm, which may result in ischemia and hypoxia in the muscle tissue and cause pelvic tissue pain. If the resting coefficient of variation is greater than 0.2, subject to unconscious contractions, suggesting a decrease in PFM stability. It causes symptoms such as painful intercourse, premature ejaculation, and constipation. Tight pelvic floor muscle groups with string-like tight bands and painful trigger points can be felt during palpation.

#### Tense contraction phase sEMG

Patients will be required to relax for 10 s and then contract for 10 s and repeat 5 times. The amplitude of the systolic wave and coefficient of variation of the slow muscle, mainly to test slow dynamic muscle strength and stability of contractile control, were assessed, with a mean sEMG of values greater than 35 μV and a coefficient of variation less than 0.2 in the normal state. When muscle strength decreases, and a coefficient of variation exceeds 0.2, symptoms such as premature ejaculation, erectile dysfunction, and urinary frequency are prone to be present.

#### Endurance contraction phase sEMG

Patients will be required to relax for 10 s and then perform an endurance contraction lasting 60 s. It aims to assess the endurance of slow muscle fiber, to test the pelvic floor muscle sEMG, especially the slow muscle fiber (normal: mean sEMG values greater than 30 μV, coefficient of variation less than 0.2). A decrease in the mean value of this stage indicates a decrease in the endurance of slow muscle fiber.

#### Post-resting EMG

Patients will be required to rest again for 1 min to relax completely. We will then test the static pelvic floor muscle tone and measure the amplitude and coefficient of variation after exercise where a value greater than 4 μV suggests elevated post-resting pelvic floor muscle tone, which can easily lead to PFM ischemia and cause clinical conditions such as painful intercourse, urinary retention, and constipation.

(2) National Institutes of Health-Chronic Prostatitis Symptom Index (NIH-CPSI).

We used a questionnaire with 13 entries, consisting of 4 aspects: pain and discomfort symptom score, urinary symptom score, symptom impact score, and quality of life score, with a total score ranging from 0 to 43. The severity of CP/CPPS can be graded according to the total scores: mild (1–14), moderate (15–29), or severe (30–43).

The efficacy evaluation criteria are developed with reference to the National Institutes of Health-Chronic Prostatitis Symptom Index score. Clinical control is defined as no less than a 90% decrease in the NIH-CPSI score, obvious improvement as no less than 60% and less than 89% decrease, improvement as no less than 30% and less than 59% decrease, and ineffective as less than 30% decrease.

#### Secondary outcome measures

1. Three-dimensional quantification and shear wave elastography (SWE).

Trans-rectal three-dimensional ultrasound (VOLUSON E8) will be used to assess the morphological parameters of the lacunae of the levator hiatus in CP/CPPS patients. The area of the levator hiatus is situated at a depth of 2–4 cm from the perineum, which is well within the effective range of 7–4-MHz transducers. The plane of minimal anteroposterior (AP) diameters will be identified in the mid-sagittal image; at this level, the axial plane will be then utilized to determine the minimum AP and lateral diameters of the levator hiatus as well as the hiatal area. After B-mode sonographic evaluation, shear wave elastography will be activated. Shear wave elastography will be used to assess the modulus of elasticity of the pubovisceral muscle (attaching laterally to the perineum) in patients with CP/CPPS. The propagation velocity of shear waves is proportional to the elastic modulus of tissue. By measuring the velocity of shear waves, the hardness of tissue can be measured directly [30]. The stiffness of the tissue will be displayed in color code rectangle.

(2) Sympathetic skin response (SSR).

SSR is a recently established method of noninvasive somatic neurography that offers the advantages of both non-invasiveness and specificity, using the SA7550 FlexComp Infiniti System (Thought Technology, Canada).

Sympathetic skin responses will be recorded with the active electrodes placed in the left palm and the reference electrodes placed on the dorsum of the left hand while the patient is in the sitting position. A channel recording from the hand will be obtained simultaneously via stimulation of the contralateral median nerve at the level of the wrist with a stimulation intensity of 20 mA for 0.2 ms [31]. Three potentials will be recorded, and the mean values will be used for analysis.

### Harm outcomes

Based on previous studies, transrectal myofascial release therapy has an approximately 15% chance of causing perianal pain, which will self-heal after two treatments and no special treatment is required.

Extracorporeal shockwave therapy has a very low probability of side effects such as edema and capillary rupture and does not require any specific treatment.

If the patient develops any discomfort during the study, or if there are any unexpected circumstances, the therapist will make a judgment and give appropriate medical treatment.

## Data collection and management

### Plans for assessment and collection of outcomes

Three groups of participants will be treated by trained therapists, and the intervention process will be strictly in accordance with the study protocol. The Primary outcome measurements will be carried out at T0, T1, T2, and T3. Secondary outcome measurements will be carried out at T0 and T3. Equipment used in the assessment, such as pelvic floor biofeedback electrostimulation, pelvic floor ultrasound, and sympathetic skin response devices, will be operated by the same researchers, and the type of equipment and the assessment site will remain the same.

### Plans to promote participant retention and complete follow-up

Once participants are enrolled in the study, the primary investigator will emphasize the importance of regular treatment for recovery. If participants can be evaluated at all stages of the treatment process, it will be free of charge. Funding will be detailed in funding statements. Participants were contacted by telephone 2 weeks after the last intervention to ensure the follow-up 4 weeks after the intervention.

### Data management and confidentiality

All data will be saved in an Easy trial database, a password-secured web-based clinical trial management system. All data is validated by having 2 individual persons enter data (double entry), and the database will check for inconsistency.

Participant data on paper obtained in the trial is kept under the participants’ ID numbers. The de-identified data will be used in the data management, and only authorized investigators will be able to access the paper documents. Publications from this trial will only contain de-identified information about trial participants. Data will not be freely available to other researchers but can be requested in a de-identified dataset to other researchers if found appropriate by the primary investigator.

### Sample size calculation

Previous studies showed that myofascial release therapy had notable effects [32] (corresponding to a Cohen’s *d* of about 0.5) on improving quality of life, prostate-related symptoms, and pain. The primary outcome will be the change in NIH-CPSI scores, the clinical response rate, and the remission rate as measured by the NIH-CPSI 4 weeks after the completed intervention. We assumed that extracorporeal shockwave combined with myofascial release therapy had similar effects on NIH-CPSI scores. A sample of 40 participants per group was computed by the G*power software to achieve a power of 60% and a level of significance of 5% and a 95% confidence level. To compensate for an estimated dropout rate of 25%, 150 participants will be ultimately required. (i.e., the number in each group is 50).

### Patient and public involvement statement

After signing the informed consent, the patient will take part in the study as a subject. Patients should receive regular treatment and assessment according to informed consent.
The research questions and outcome measures are developed according to the possible pathogenesis and previous studies.
3.No form of recruitment was undertaken for this study.
Patients and the public will not be involved in the design of this study.
Patients and the public will not be asked to assess the burden of the intervention and time required to participate in the research.
No recruitment advertisements will be published. Patients, who plan to receive the treatment, will be included in this study.
The results will be published in a peer-reviewed journal upon study completion.

### Blinding

This study was a double-blinded, randomized, controlled trial. The subjects had no knowledge of the distribution. Therapists will not be involved in collecting data, and data managers will not participate in the intervention of patients.

#### Extracorporeal shockwave pseudo-therapy

A plastic film is placed over the treatment head, so the ultrasound waves cannot act on the treatment area.

#### Myofascial release group pseudo-therapy

Apply pressure only to pain-free points on the patient’s abdomen, using gentle movements to produce as little pain as possible.

### Statistical methods

Statistical analysts perform data analysis in a blind state. All statistics analyses will be performed using SPSS 25.0 for Windows (SPSS Inc., Chicago, IL, USA). All data will be entered into SPSS by the research assistant. A *p*-value of < 0.05 will be considered statistically significant. One-way (3 groups: single extracorporeal shockwave therapy vs single myofascial release therapy vs combined therapy) analysis of variances (ANOVA) with repeated measures will be performed to compare changes in sEMG assessment and NIH-CPSI scores between and within groups over different time points (i.e., T0-T2). A paired sample *t*-test was used to compare the differences in outcome indicators between groups at T2 and T3 to determine the long-term efficacy of each intervention. Post hoc analyses will be performed when any significant difference both between- and within-group is found in any of the outcome variables. Bonferroni correction will be used to adjust the alpha levels. Multilevel regression or a generalized estimating equation (GEE) will also be used as a sensitivity analysis to analyze the correlation between NIH-CPSI scores and the results of sEMG assessment of the pelvic floor muscle and the results of sympathetic skin response. Mediation analysis will examine the mediating effect of sEMG assessment at T1 on the relation between extracorporeal shockwave therapy and NIH-CPSI scores at T2. This study strictly controlled for gender, age, and BMI of the participants, which may affect the primary and secondary outcomes. The mean absolute difference (MAD) and standard error of measurement (SEM) will be used to evaluate inter-method reliabilities. Consequently, Bland–Altman plots will be used to investigate the agreement between NIH-CPSI and SSR. Since the two measurement results have different measurement units, a *Z*-score will be calculated based on the formula: *Z*-score = (observation value − mean value)/SD.

## Discussion

CP/CPPS is often misdiagnosed as prostatitis and is commonly treated with antibiotics. However, in recent years, some studies have shown that pharmacological treatments do not have a significant effect and maybe even no difference from placebo [33, 34]. The pathogenesis of CP/CPPS is currently unknown. However, studies on this have mainly shown an association with the following mechanisms: (1) inflammation of the pelvic organs (prostate, bladder, uterus) and/or PFM leads to increased expression of several immunologically active mediators that act via neuronal crosstalk on the corresponding tissues, adjacent organs, and the central nervous system [35, 36]; (2) overuse and abnormal postures will activate the myofascial and thus produce permanent contraction that produces pain [37] and increase the tension even in the most relaxed state; this will prevent full elongation of the muscle [38] and thus limit the range of motion to some extent [39]; (3) inflammation within the prostate causes irritation of the prostate and peripheral nervous system in sensitive individuals and induces some pain [40]; and (4) long-term fear, tension, depression, stress, and other mental factors will lead to systemic plant nerve dysfunction [41], which increases alpha1 receptor excitability. Then, this dysfunction will cause neuromuscular dysfunction in the posterior urethra and increase urethral pressure, making it easier for urine to reflux and creating a sense of incomplete urination. One of our purposes is to determine the association between CP/CPPS symptoms and sympathetic nerves in order to explain the pathogenesis.

It is now recommended by the British Urology Association that the main aim of CP/CPPS treatment is to mitigate functional damage to patients [42], usually with single internal and external fascia manipulation. However, most of the studies on myofascial release therapy are pre-and post-controlled trials while lacking high-quality randomized controlled trials [43]. As mentioned in the literature review, myofascial release therapy can effectively alleviate the symptoms of pelvic floor pain in patients, but evidence for the intervention efficacy remains greatly inconclusive because the main primary outcome in existing studies is subjective scale [32, 44, 45]. At present, there are no studies on myofascial release therapy combined with extracorporeal shockwave therapy. Our study will compare three treatments—single myofascial release therapy, extracorporeal shockwave therapy, and combined therapy—and then propose to compare the relationship between the three treatment modalities and the subjective and objective functional changes before and after treatment.

In previous assessments, we found that the symptoms of chronic pelvic pain syndrome may be closely associated with increased pelvic floor muscle tone, which can be effectively reduced with myofascial release therapy. In this study, by comparing the association between the primary and secondary outcome measure and different therapy modalities (extracorporeal shockwave, myofascial release, and combined therapy) on the therapeutic effect of pelvic floor hypertonia at T0-T3 stage, it was proposed to determine (1) if shockwave therapy can reduce pelvic floor muscle tension further after taking myofascial release therapy; (2) if the combined therapy can reduce pelvic floor muscle tone, relieve the discomfort symptoms, and improve the quality of life of the patients more efficiently and have a long-term effect than single treatment [46, 47]; (3) if curative effect can be reflected by the change of sympathetic skin response before and after treatment; and (4) if there exist other mechanisms that may improve the symptoms of patients other than what extracorporeal shockwave and myofascial release therapy did on reducing pelvic floor muscle tone.

This study innovatively uses the modulus of elasticity to assess pelvic floor muscle tone, which has previously been used to assess the muscle status of spastic muscles in hemiplegic patients with good sensitivity [48, 49]. In the course of the previous treatment, it was found that the pelvic floor muscles became less stiff and more elastic after myofascial release therapy, but the improvement in elasticity has not been assessed by objective criteria.

Sympathetic skin response is a transient potential change in the skin that is triggered by endogenous or exogenous stimuli. As a noninvasive test, SSR can be used clinically to detect disorders related to sympathetic tone [50]. Due to the extensive distribution of sympathetic postganglionic fibers in the skin and the fact that the nerves in the skin of the upper limbs and chest originate from the cervical and stellate ganglia, stellate ganglion nerve activity can be reflected by upper limb skin sympathetic nerve activity. Since reduction of CP/CPPS symptoms can significantly improve depression, anxiety, and pain symptoms of patients [16], we presume the possible pathogenesis of chronic pelvic pain syndrome may be the altered sympathetic nerve function caused by anxiety and depression.

This study will compare the agreement between patients’ sympathetic skin response and NIH-CPSI scores at different stages of treatment. If the results of this study show good linear correlations, it may suggest that one of the pathogenic mechanisms of CP/CPPS is hypo- or hyper-autonomic function and that more effective combination treatments could be developed by focusing on this pathogenesis in the future. This proposed study aims to contribute to this growing area of research by exploring the pathogenesis and novel therapeutic targets of CP/CPPS.

Moreover, the study may yield a limitation: we could only realize that participants and evaluators were blinded, and the therapist would know the grouping. We will try to improve our method in the follow-up research, trying to achieve the requirement that therapists be blinded.

## Trial status

Protocol version 20,220,710, 1 August 2022. The trial is currently in the process of recruiting participants in Zhongda Hospital Affiliated to Southeast University and is expected to be completed in June 2024.

### Supplementary Information

**Additional file 1.** SPIRIT checklist.

**Additional file 2.** NIH-CPSI.

**Additional file 3.** Model consent form.

## Data Availability

No datasets were generated or analyzed during the current study. The results will be published in a peer-reviewed journal upon study completion. The corresponding author and IEC (Independent Ethics Committee) for Clinical Research of Zhongda Hospital will have access to the final trial dataset. Datasets generated or analyzed in this study will be provided by the corresponding authors upon reasonable request.

## Abbreviations

CP/CPPS: Chronic prostatitis/chronic pelvic pain syndrome
Li-ESWT: Low-intensity extracorporeal shockwave therapy
ESWT: Low-intensity extracorporeal shockwave therapy
NIH-CPSI: National Institutes of Health-Chronic Prostatitis Symptom Index
HRQoL: Health-related quality of life
PFM: Pelvic floor muscle
ANS: Autonomic nervous system
BMI: Body mass index
ANOVA: Analysis of variances
sEMG: Surface electromyography
AP: Anteroposterior
SSR: Sympathetic skin response
GEE: Generalized estimating equation
MAD: Mean absolute difference
SEM: Standard error of measurement
