# Supplementary material for: Comparing the effectiveness of extracorporeal shockwave therapy and myofascial release therapy in chronic pelvic pain syndrome: study protocol for a randomized controlled trial
Source: Trials. 2023 Oct 18;24:675. doi: 10.1186/s13063-023-07633-1 (PMC10583345; doi:10.1186/s13063-023-07633-1)
Supplement: Supplementary file 2 — Additional file 2. NIH-CPSI. [file 13063_2023_7633_MOESM2_ESM.docx]

| NIH-Chronic Prostatitis Symptom Index (NIH-CPSI) | | |
| --- | --- | --- |
| Pain or Discomfort | NO | YES |
| 1.In the last week, have you experienced any pain or discomfort in the following areas? | | |
| 1. Area between rectum and testicles (perineum) | ❑_0_ | ❑_1_ |
| 1. Testicles | ❑_0_ | ❑_1_ |
| 1. Tip of the penis (not related to urination) | ❑_0_ | ❑_1_ |
| 1. Below your waist, in your pubic or bladder area | ❑_0_ | ❑_1_ |
| 2.In the last week, have you experienced: | | |
| 1. Pain or burning during urination? | ❑_0_ | ❑_1_ |
| 1. Pain or discomfort during or after sexual climax (ejaculation)? | ❑_0_ | ❑_1_ |
| 3.How often have you had pain or discomfort in any of these areas over the last week? | | |
| ❑_0_ Never ❑_1_ Rarely ❑_2_ Sometimes ❑_3_ Often ❑_4_ Usually ❑_5_ Always | | |
| 4. Which number best describes your AVERAGE pain or discomfort on the days that you had it, over the last week? | | |
| ❑ ❑ ❑ ❑ ❑ ❑ ❑ ❑ ❑ ❑ ❑  0 1 2 3 4 5 6 7 8 9 10  No pain as pain as  you can imagine | | |
| Urination | | |
| 5. How often have you had a sensation of not emptying your bladder completely after you finished urinating, over the last week? | | |
| ❑_0_ ❑_1_ ❑_2_  Not at all Less than 1 time in 5 Less than half the time  ❑_3_  ❑_4_ ❑_5_  About half the time More than half the time Almost always | | |
| 6. How often have you had to urinate again less than two hours after you finished urinating, over the last week? | | |
| ❑_0_ ❑_1_ ❑_2_  Not at all Less than 1 time in 5 Less than half the time  ❑_3_ ❑_4_ ❑_5_  About half the time More than half the time Almost always | | |
| Impact of Symptoms | | |
| 7. How much have your symptoms kept you from doing the kinds of things you would usually do, over the last week? | | |
| ❑0 None ❑1 Only a little ❑2 Some ❑3 A lot | | |
| 8. How much did you think about your symptoms, over the last week? | | |
| ❑0 None ❑1 Only a little ❑2 Some ❑3 A lot | | |
| Quality of Life | | |
| 9. If you were to spend the rest of your life with your symptoms just the way they have been during the last week, how would you feel about that? | | |
| ❑_0_ Delighted ❑_1_ Pleased ❑_2_ Mostly satisfied ❑_3_ Mixed ❑_4_ Mostly dissatisfied ❑_5_ Unhappy ❑_6_ Terrible | | |

Fig.2 Healing Index n=(Pre-treatment NIH-CPSI score - Post-treatment NIH-CPSI score) / Pre-treatment NIH-CPSI score × 100%

Scoring the NIH-Chronic Prostatitis Symptom Index

Domains Pain: Total of items 1a, 1b, 1c,1d, 2a, 2b, 3, and 4 =

Urinary Symptoms: Total of items 5 and 6 =

Quality of Life Impact: Total of items 7, 8, and 9 =
