# Supplementary material for: Comparing the effectiveness of extracorporeal shockwave therapy and myofascial release therapy in chronic pelvic pain syndrome: study protocol for a randomized controlled trial
Source: Trials. 2023 Oct 18;24:675. doi: 10.1186/s13063-023-07633-1 (PMC10583345; doi:10.1186/s13063-023-07633-1)
Supplement: Supplementary file 3 — Additional file 3. Model consent form. [file 13063_2023_7633_MOESM3_ESM.docx]

知情同意书

体外冲击波联合筋膜手法治疗慢性盆腔疼痛综合征的干预研究

知情同意书·知情告知页

尊敬的女士/先生：

我们将邀请您参加一项体外冲击波联合筋膜手法治疗慢性盆腔疼痛综合征的干预研究。本研究已通过东南大学附属中大医院临床研究伦理委员会审查批准（批件号： ）。

在您决定是否参加这项研究之前，请尽可能仔细阅读以下内容。它可以帮助您了解该项研究以及为何要进行这项研究，研究的程序和期限，参加研究后可能给您带来的益处、风险和不适。如果您愿意，您也可以和您的亲属、朋友一起讨论，或者请医生给予解释，帮助您做出决定。

1. **研究背景、研究目的和研究方法**

**1.本研究背景**

既往研究证明，体外冲击波治疗（ESWT）作为一种物理治疗方式，在慢性前列腺炎/慢性盆腔疼痛综合征（chronic prostatitis/chronic pelvic pain syndrome，CP/CPPS）患者的NIH-CPSI（[美国国立卫生研究院慢性前列腺炎症状指数](http://www.baidu.com/link?url=jGAkAYCC72MNXVVsq6QicY1-d-jFmqUXuh4ElvWREuUHc7cfnDatci2SkF5drkf-ChKHQulxjhI2ddk1JMgc0Mb37JfiGRZ03eXcoifmWZG)）评分、疼痛和生活质量等方面有改善作用。ESWT通过利用某些组织类型中的机械转导系统来调节各种细胞和分子机制。作用于局部组织，ESWT可以增强血管内皮生长因子（VEGF）及其受体Flt-1的表达，并能诱导新生血管形成，改善心肌缺血；通过体外冲击波治疗可改善阴茎局部血流供应，从而改善勃起功能，且疗效较为持久稳定。基于此，通过体外冲击波治疗患者张力异常增高的盆底肌，可能促进局部血管生成，改善血液循环，促进致痛物质代谢，最终减轻患者疼痛不适感。

筋膜手法治疗是指对盆腔内部及外部筋膜进行的手法治疗，已被证明能有效缓解CP/CPPS患者症状，筋膜手法治疗基于肌筋膜具有整体性且筋膜间存在力的传递等现实依据，涉及对肌筋膜复合体施加可变负荷和长时间拉伸，旨在恢复筋膜最佳长度、恢复张力平衡、减轻疼痛和改善功能。

体外冲击波联合筋膜手法治疗慢性盆腔疼痛综合征，从改善整体血供和缓解局部痉挛两方面，减轻CP/CPPS患者症状，较传统治疗方式相比，疗效可能更持久且更有针对性。

**2.本研究目的**

比较慢性盆腔疼痛综合征患者治疗前后的NIH-CPSI评分（包括疼痛和不适症状评分、排尿症状评分、症状影响评分、生活质量评分4各方面）、盆底肌电、盆底超声、血流动力等方面的特征性指标，确定冲击波联合筋膜手法治疗慢性盆腔疼痛综合征的有效性，为慢性盆腔疼痛综合征的评估、治疗提供依据。寻找针对慢性盆腔疼痛综合征更为有效的干预措施。

**3.研究设计**

3.1研究方法

文献资料法：针对课题的需要，在东南大学图书馆査阅相关文献，并通过中文期刊网、中国知网、PUBMED、Web of Science检索相关文献，进行归纳总结，借鉴文献成果为本课题提供理论依据。

数理统计法：对收集的问卷及影像资料进行整理，运用spss.27.0和EXCEL2003软件系统对回收的问卷进行统计学处理与分析，最终结果经过多次确认无误后，用于本研究工作的分析和论证。

逻辑分析法：将主观量表、表面肌电、超声检查所获得的数据与事实进行逻辑思辩，对收集到的资料进行归纳、分析，并结合以往的研究成果运用归纳和演绎的方法得出本课题的结论。

3.2研究参加单位和预计纳入受试者例数

本研究参加的单位为东南大学附属中大医院，计划纳入98例受试者，其中试验组49例，对照组49例。

3.3研究预期持续时间、项目启动时间

研究持续时间2年（2022.9.1—2024.9.1）

项目启动时间：2022年9月1日

3.4受试者的主要纳入标准

①根据美国国立卫生研究院（NIH）的实际分类，所有男性受试者均符合慢性前列腺炎IIIB型/慢性骨盆疼痛综合征的诊断标准（膀胱、腹股沟、会阴区、生殖器或下腹部疼痛，泌尿学检查无明显异常）。②年龄20-40岁；③持续3个月以上的慢性盆腔疼痛,疼痛区域VAS评分大于3分；④签署知情同意书。

3.5受试者的主要排除标准

①有显著的凝血障碍、会阴解剖异常、雄激素异常以及神经系统异常者；②有明确盆腔疼痛病因者；③研究开始时接受其他治疗；④存在任何尿道病变者；⑤伴有其他导致盆腔疼痛疾病的患者；

**二、受试者责任**

1． 在您入选研究前，研究者将询问、记录您的病史，并进行疼痛、前列腺血流动力、肛提肌厚度及面积、肛提肌裂孔面积、前列腺症状障碍指数、盆底表面肌电检查。

您是合格的纳入者，您可自愿参加研究，签署知情同意书。

如您不愿参加研究，我们将遵循您的意愿。

2. 若您自愿参加研究，将按以下步骤进行：

受试者需要遵守的试验步骤，包括创伤性医疗操作。

2.1 基础触诊

所有受试者都经过康复治理师的物理检查，以确定肌肉中的压痛点和触发点。检查包括对外部和内部肌肉的触诊。受试者仰卧和侧卧，触诊腹壁、背部、臀部和大腿的肌肉。在受试者截石位进行直肠内触诊。记录受试者激痛点数量、疼痛程度、疼痛性质。

2.2 对照组筋膜手法治疗

根据触诊结果，对受试者疼痛评分均在1分以上的触诊点持续按压，按压1 kg/cm2(在受试者可耐受范围内因人而异)。持续按压直到肌肉松弛，按压时间为180~210s。所有受试者均接受筋膜手法治疗，每周2次，连续治疗4周。

2.3 试验组在筋膜手法治疗基础上配合体外冲击波治疗

接受冲击波治疗的受试者在截石位接受会阴部体外冲击波（ESWT）治疗，每周2次，持续4周，每次3000次脉冲。对照组不接受体外冲击波治疗的受试者接受相同设备的相同治疗头，但治疗头带有一层充气微球薄膜以吸收冲击波，作为安慰剂不起到治疗效果。

2.4 随访时间

受试者须在8次治疗后再次进行疼痛、前列腺血流动力、肛提肌厚度及面积、肛提肌裂孔面积、前列腺症状障碍指数、盆底表面肌电检查，并在治疗结束后第4、8周参与线上问卷随访。

在上述治疗/检查中，聚焦式体外冲击波治疗是研究性的，如果您不参加本研究，就不需要接受体外冲击波联合筋膜手法治疗慢性盆腔疼痛综合征的干预研究项目的治疗。

3. 需要您配合的其他事项

您需要按医生和您约定的随访时间来医院就诊。您的随访非常重要，因为医生将根据随访评价研究措施的作用。在研究期间您不能使用治疗慢性前列腺炎/慢性盆腔疼痛综合征（CP/CPPS）的其它药物(或其他对本研究造成影响的治疗手段)。

如您需要进行其它治疗，请事先与您的医生取得联系。

三、受试者权益

您参加研究是自愿的，参加研究的信息是保密的，您可以拒绝参加该研究或者在任何时候退出该研究都不会遭到歧视或报复，您的医疗待遇和权益不会受到影响。

您可以选择不参加本项研究，或中途退出研究，您可向研究者询问有可能获得的备选治疗方法，包括筋膜手法治疗、盆底磁刺激、生物反馈电刺激治疗。您不必为了治疗您的疾病而必须选择参加本项研究。

如果您需要其他治疗，或者您没有遵守研究计划，或者有任何其他合理原因，您的医生或研究者出于对您的最大利益考虑，可能会随时中止您参加本项研究。

如果您因为任何原因从研究中退出，您可能被询问有关您使用试验药物的情况。如果医生认为需要，您也可能被要求进行试验室检查和体格检查。这对保护您的健康十分有利。

如果在研究过程中有任何重要的新信息，可能影响您继续参加研究的意愿时，您的医生将会及时通知您或您的监护人。

您可随时了解与本研究有关的信息资料和研究进展，如果您有与本研究有关的问题，或您在试验过程中发生了任何不适与损伤，或有关于本研究涉及您权益方面的问题，您可以随时向研究者进行咨询。

研究者姓名： 孙武东 联系方式： 13813900191

如果您对参加本研究有任何抱怨，请联系东南大学附属中大医院临床研究伦理委员会，联系方式：025-83272015。

**四、参加研究可能的受益**

该受益包括您的病情有可能获得改善，以及本项研究可能帮助开发出一种新治疗(检查或者其他医疗目的相关的)方法，以用于患有相似病情的其他病人。

您也可能不能从该项目中获益，因为传统筋膜手法治疗有极低的可能无法缓解CP/CPPS患者症状。

**五、参加研究可能的不良反应、风险和不适、不方便**

经直肠筋膜手法治疗有15％左右的可能性引起肛周疼痛，一般治疗2次后自行缓解，无需特殊处理，治疗后期无其他明显不良反应。

体外冲击波在极低的概率下具有副作用，如：局部水肿、毛细血管破裂等，无需特殊处理。

您在研究期间需要按时到医院随访，做一些检查，这将占用您的一些时间，也可能给您造成麻烦或带来不便。

如果在研究期间您出现任何不适，或病情发生新的变化，或任何意外情况，不管是否与研究有关，均应及时通知您的医生，他/她将对此作出判断并给与适当的医疗处理。

**六、有关费用**

参加该研究您不会获得任何报酬，同时也不会收取您任何费用。

**七、有关补偿**

研究者将尽全力预防和治疗由于本研究可能带来的伤害。如果在研究中出现与研究相关的损害时，将按照我国《药物临床试验质量管理规范》的规定提供治疗的费用及相应的经济补偿。

**八、个人信息的保密**

您的医疗记录及资料将完整地保存在医院。研究者、伦理委员会和药品监督管理部门、卫健委管理部门将被允许查阅您的医疗记录。任何有关本项研究结果的公开报告将不会披露您的个人身份。我们将在法律允许的范围内，保护您个人医疗资料的隐私。

按照医学研究伦理学原则，除了个人隐私信息外，试验数据将可供公众查询和共享，查询和共享将只限于基于网络的电子数据库，保证不会泄漏任何个人隐私信息。

除本研究以外，有可能在今后的其他研究中会再次利用您的医疗记录和病理检查标本。您现在也可以声明拒绝除本研究外的其他研究利用您的医疗记录和病理检查标本。

知情同意书.同意签字页

**同意声明**

我已经阅读了上述有关本研究的介绍，而且有机会就此项研究与研究者讨论并提出问题。我提出的所有与研究相关的问题均得到答复，同时我与我的家人有充足的时间对此进行考虑。

我知道参加本研究可能产生的风险和受益。我知晓参加研究是自愿的，而且明白：

- 该研究已通过东南大学附属中大医院临床研究伦理委员会批准。
- 我所有的信息是保密的。
- 我的隐私权、医疗和补偿权得到了保障。
- 我可以随时向研究者咨询更多的信息。
- 我可以选择不参加本研究，或者可以随时退出本研究，而不会受到歧视或报复，我的医疗待遇与权益不会受到影响。
- 如果我中途退出研究，特别是由于药物的原因使我退出研究时，我应将我的病情变化告诉研究者，完成相应的体格检查和理化检查，这将对整个研究十分有利。
- 如果因病情变化我需要采取任何其他的治疗，或者我没有遵守研究计划，我会在事先征求研究者的意见，或在事后如实告诉研究者，研究者可以因此或者因为其他合理原因终止我继续参加本研究。

我同意药品监督管理部门、卫健委管理部门、伦理委员会或申办者代表查阅我的研究资料。

我将获得一份经过签名并注明日期的知情同意书副本。

最后，我决定同意参加本项研究，并保证尽量遵从研究程序。

**我同意□ 拒绝□ 除本研究以外的其他研究利用我的医疗记录和病理检查标本。**

受试者签名： 日期： 年 月 日 时 分

联系电话：

监护人签名（如适用）： 日期： 年 月 日 时 分

受试者姓名（正楷）： 监护人与受试者的关系：

监护人联系电话：

公正见证人签名（如适用）： 日期： 年 月 日 时 分

公正见证人联系电话： 公正见证人身份证号码：

我确认已向受试者解释了本研究的详细情况，包括其权力以及可能的受益和风险，，并对其提问进行了解答，受试者自愿参加该研究，已给其一份签署过的知情同意书副本。

研究者签名： 　 日期： 年 月 日 时 分

研究者联系电话：
